# Patterns and Risks of China’s Snake Trade Driven by Medicinal and Culinary Traditions

**DOI:** 10.3390/ani16111624

**Published:** 2026-05-27

**Authors:** Xiang-Mo Li, Shan Su, Lu-Wen Zhang, Yan-Qing Wu, Xiang Ji

**Affiliations:** 1 Herpetological Research Center, College of Life Sciences, Nanjing Normal University, Nanjing 210023, China; lixiangmo0831@outlook.com (X.-M.L.); susan940906@163.com (S.S.); 2 Zhejiang Provincial Key Laboratory for Water Environment and Marine Biological Resources Protection, College of Life and Environmental Sciences, Wenzhou University, Wenzhou 325035, China; yanqingwu@wzu.edu.cn; 3 School of Life Sciences, East China Normal University, Shanghai 200241, China; zhangluwen1022@163.com

**Keywords:** China, CITES, medicinal food culture, misreported species, snake trade, venomous snakes, wildlife consumption

## Abstract

China plays a multifaceted and pivotal role in the global snake trade network, with its trade dynamics heavily influenced by domestic market demand and international regulatory policies. Based on official CITES trade data from 1975 to 2023, this study systematically analyzes the scale, species composition, source dynamics, and potential risks of China’s legal snake trade by using Whole Organism Equivalents (WOEs) and trade measures adjusted for reporting effort. The results show that the cumulative trade volume exceeded 11.7 million WOEs and that China’s role underwent a major transformation from a “supply center” in the early 1990s to a “consumption hub” by 2012. Export patterns were dominated by colubrid snakes (81.5% of total exports), with the oriental ratsnake (*Ptyas mucosa*) alone accounting for 56.8%; this reflects a distinctive trade landscape shaped by the domestic culture of “medicine and food homology.” Although the proportion of captive-bred individuals has increased since 2010, a dual-supply system comprising both wild-sourced and captive-bred individuals persists. Furthermore, we identified several misreported species, exemplified by the non-native common cobra *Naja naja* being recorded as wild-sourced exports from China. Notably, the live venomous snake trade exhibits an extreme geographic concentration, with over 93% of exports directed to Hong Kong, thereby posing significant biosecurity and public health risks. In conclusion, future governance should move beyond simple trade bans by utilizing molecular tools to correct identification biases and by implementing regulatory frameworks modeled on the Hazard Analysis Critical Control Point (HACCP) system. This approach will address biosecurity risks within the trade chain and help achieve a scientifically informed balance between ecological conservation and livelihood security.

## Simple Summary

By analyzing the CITES (Convention on International Trade in Endangered Species of Wild Fauna and Flora) trade data from 1975 to 2023, we provide a comprehensive portrait of China’s temporal dynamics and its conservation implications within the global snake trade network. China, together with the United States and Japan, ranks among the top three legal wildlife trading nations globally, serving as a global processing and export center for snake skin products and a critical hub for the live snake trade, particularly for venomous species. This pattern, driven by a robust domestic culinary and medicinal market, has exhibited remarkable stability over the past half-century. This sustained and concentrated trade pressure has become a core driver of species depletion, suggesting that habitat protection alone is no longer sufficient. There is an urgent need for more stringent trade regulations and scientifically rigorous sustainability assessment frameworks. Future governance should adopt an integrated strategy that explicitly couples controlled captive breeding with sustainable wild population management and habitat conservation goals. This requires the establishment of mandatory provenance audits and traceability systems, the promotion of standardized farming certifications, and the implementation of humane slaughter standards.

## 1. Introduction

International wildlife trade is a multi-billion-dollar global industry that profoundly impacts the planet’s biodiversity [1]. The scale of this trade is striking: an estimated 24% of terrestrial vertebrate species are affected by trade activities [2,3]. Trade acts as a direct driver of population depletion, driving, on average, a 62% decline in the abundance of traded species [2]. Within the ongoing biodiversity crisis, over 35% of reptilian species globally are involved in the online trade [4]. Among these trade-affected species, approximately 90% of the species and half of the individual specimens originate directly from wild-sourced captures [4]. Snakes, in particular, have become primary targets for both legal and illegal trade due to their extensive use as food [5], in traditional medicine [6], for luxury leather [7], and in the pet industry [8], facing immense harvest pressure. Such exploitation not only exerts devastating impacts on wild populations [9] but also risks the introduction of invasive alien species and pathogens, posing threats to native ecosystems and public health [10,11].

To address these challenges, the Convention on International Trade in Endangered Species of Wild Fauna and Flora (CITES) serves as the primary international regulatory instrument. Although the CITES database faces significant systemic limitations in species coverage [4,12], multi-source data consistency [3], and the representation of illegal trade [1], it remains the most authoritative and comprehensive data source for analyzing and understanding the legal international trade patterns of regulated species. In this complex global context, China’s role is particularly pivotal as one of the world’s largest wildlife consumer markets. Unlike the global high-end leather market, which is primarily driven by python skins [13], China’s snake trade exhibits a distinctive pattern shaped by vast domestic demand, spanning the culinary, medicinal, and leather sectors [14]. In recent years, captive breeding has been widely proposed as a solution to alleviate pressure on wild populations; however, its conservation efficacy remains controversial, as it may facilitate the ‘laundering’ of illegally sourced wild individuals [15,16].

Therefore, this study aims to fill these existing knowledge gaps by analyzing nearly half a century of official CITES data from 1975 to 2023. Our specific objectives are to (1) describe and quantify the overall scale, species composition, and dynamic trends of China’s legal snake trade; (2) analyze the transition of trade sources from wild-sourced capture to captive breeding; (3) identify key anomalies and inconsistencies within the trade data; and (4) map the geographic patterns of China’s role in the global snake trade network, with a particular focus on the live venomous snake trade, to evaluate its central position. The results of this study will provide a robust evidence base for understanding China’s complex role within the broader landscape of global wildlife trade and will offer scientific foundations for developing more targeted and effective species conservation and trade management policies.

## 2. Materials and Methods

Data for this study were extracted from the CITES Trade Database (1975–2023), which is managed by the UN Environment Programme World Conservation Monitoring Centre (UNEP-WCMC) on behalf of the CITES Secretariat. This database compiles official annual trade reports submitted by its 185 signatory parties. Queried variables included Year, Importer, Exporter, Trade Term, Source, Purpose, and Taxon. Species conservation status was determined by consulting the IUCN (International Union for Conservation of Nature) Red List of Threatened Species, while taxonomic nomenclature, legislative status, and distribution data were cross-referenced via the Species+ platform.

The taxonomic framework for snakes primarily follows that of the Reptile Database [17]. To ensure taxonomic currency, outdated scientific names were updated to reflect the latest classifications (Table S1), and all subsequent statistical analyses were conducted using these revised names.

Despite data gaps in certain years (e.g., 1982–1985), these periods were retained in the analysis to maintain temporal continuity. Given that the number of CITES-listed species and signatory parties fluctuates over time, we included species that were listed either historically or currently in CITES Appendices. Since these fluctuations directly influence trade reporting coverage, relying solely on raw trade volumes may introduce bias and obscure underlying trends. To account for reporting bias, we present both unadjusted raw trade data and standardized data that are corrected for reporting effort. Following Hierink et al. (2020) [18], a yearly reporting effort metric was calculated as the number of CITES parties multiplied by the number of listed snake species for each year. Raw trade quantities were then divided by this correction factor to obtain standardized trade estimates for each year. To exclude the influence of regional trade regulations (e.g., EU annexes), only species listed in the CITES Appendices (historically or currently) were included.

Trade volumes are expressed as Whole Organism Equivalents (WOEs), following the methodology of Harfoot et al. (2018) [19]. WOEs incorporate trade terms such as “live,” “bodies,” “skins,” “gall bladders,” “skulls,” “specimens,” “tails,” “heads,” “trophies,” and “skeletons.” We assume each record represents one individual and that separate products (e.g., a head and a gall bladder) each originates from different individuals. While this assumption may slightly overestimate actual trade volumes, the resulting bias is considered minimal. Quantities were aggregated when units were ambiguous. Trade in live snakes was isolated separately to assess potential biosecurity and health risks. Terms that could not be converted to WOEs—such as derivatives (e.g., oils, gels), footwear, and leather products—were excluded from the analysis [accounting for 23.0% of import records (*n* = 1,368,817) and 46.0% of export records (*n* = 6,123,113) in China].

CITES trade records can be reported by either the importer or the exporter. Where feasible, results from both reporting sides are presented; however, for specific calculations (e.g., percentages), exporter-reported data were used in preference. This accounts for the potential systematic underestimation in importer reports, as parties are not required to issue import permits for CITES Appendix II species, and these species account for 74.1% of China’s total WOE export records [20].

To distinguish between direct trade and re-exports, we followed the criteria established by the UNEP-WCMC and Robinson and Sinovas (2018) [20]. Re-exports were defined as records for which the “Origin” field was populated or contained an origin permit ID. Conversely, direct trade refers to records for which the “Origin” field remained blank, and there was no origin permit ID. To avoid double-counting, re-exports were excluded from most analyses, except for the analysis of total WOEs, in which they were retained to fully reflect species consumption across the trade chain.

The comparative analysis of wild-sourced versus captive-bred snakes was based on a reclassification of the “Source” variable, following Hierink et al. (2020) [18]. Source codes for “Wild-caught” (W), “Unknown” (U), “Taken from the marine environment” (X), and “Ranched” (R) were grouped into the Wild-sourced category. Codes for “Captive-bred” (C and D), “Artificially propagated” (A), and “Born in captivity” (F) were grouped into the “Captive-bred” category (Table S2).

Spatial analysis involved matching two-letter ISO country codes (ISO2) with full CITES party names. To highlight major trade patterns and minimize background noise from small-scale shipments, we applied cut-off thresholds. For live venomous snakes, the thresholds were categorized as 0–5000 (blue), 5001–10,000 (orange), and >10,000 (red) individuals.

## 3. Results

### 3.1. Scale, Structure, and Dynamics of China’s Snake Trade

Between 1975 and 2023, China’s cumulative snake trade exceeded 11.7 million Whole Organism Equivalents (WOEs). The overall pattern exhibits a distinct export-oriented characteristic, with total exports (7.174 million WOEs) approximately 1.5 times total imports (4.596 million WOEs) (Table 1). The trade flows show high taxonomic concentration: snakes of the family Colubridae exert absolute dominance, accounting for 81.5% of total exports and 53.5% of total imports. Notably, the oriental ratsnake (*Ptyas mucosa*) is the core trade species, accounting for 42.0% of imports and 56.8% of exports (Table 2). In contrast, the structure of imports and exports reveals significant family-level differences: while exports are primarily driven by Colubridae and Elapidae, imports show a heavy reliance on Pythonidae (26.6% of total imports), with species such as the reticulated python (*Malayopython reticulatus*) and the Burmese python (*Python bivittatus*). Furthermore, approximately 74.1% of exported individuals belong to CITES Appendix II species, reflecting the extensive international regulatory oversight applied to this trade.

**Figure 1:**
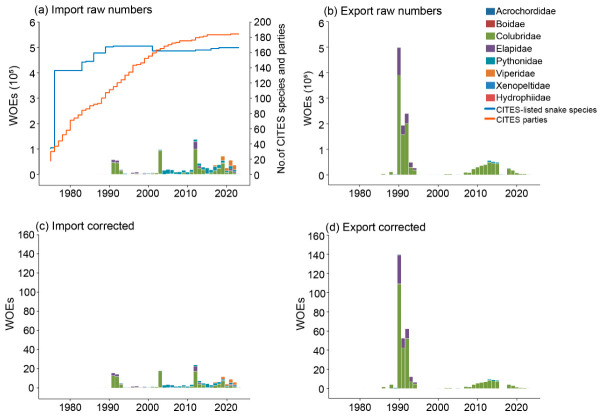
Temporal dynamics in whole organism equivalents (WOEs) for snake imports (**a**,**c**) and exports (**b**,**d**) in China, categorized by snake family. The cumulative number of CITES parties (orange line) and the number of snake species listed in CITES (blue line) are given in Plot (**a**).

In terms of trade forms and temporal dynamics, skins are the dominant commodity, accounting for 89.1% of total exports and 53.3% of total imports. Live trade also represents a significant share of imports (35.5%), primarily comprising colubrid snakes (Table 1). Beyond individual specimens, China is involved in a large-scale snake meat trade, with imports (1.163 million kg) far exceeding exports (0.221 million kg), indicating robust domestic consumption demand. Regarding temporal dynamics, China’s snake trade exhibited significant fluctuations over the study period. Export peaks were concentrated in the late 1990s and mid-2010s. Imports, however, displayed a multi-peak pattern (early 1990s, 2003, 2012, and 2019), driven by alternating demands for Colubridae, Elapidae, and Pythonidae (Figure 1). Although commercial purposes remain the primary driver of trade, there is a notable lack of purpose-related information in the records (cumulatively approximately 2.5 million WOEs), posing challenges for accurately assessing trade motivations and regulatory effectiveness (Table S3).

### 3.2. Source Composition, Conservation Status, and Geographical Patterns

**Figure 2:**
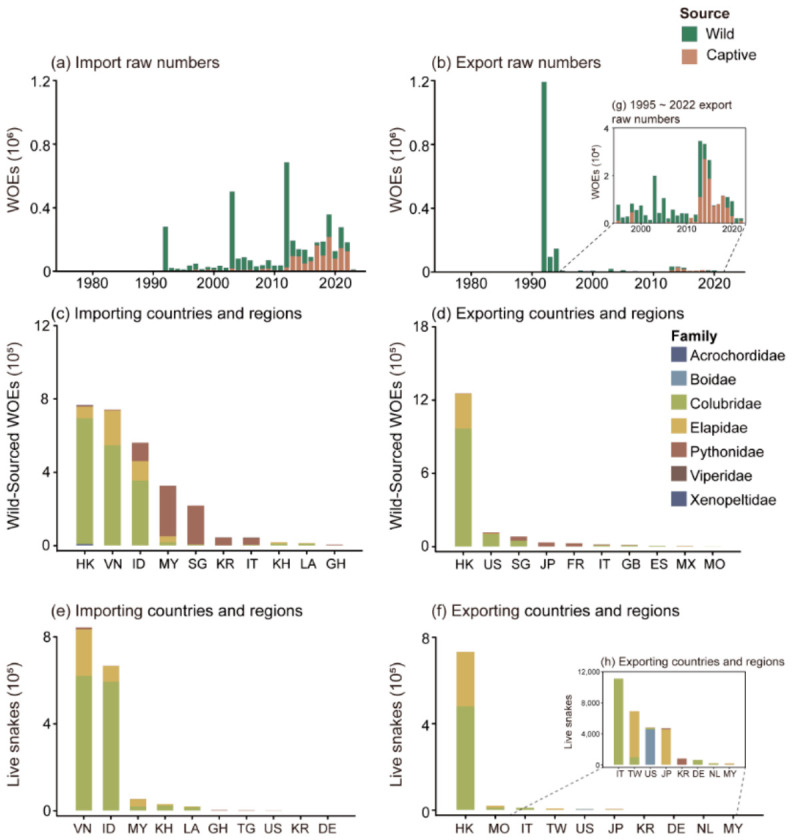
Spatiotemporal patterns and taxonomic composition of the international snake trade in China. Annual trends in the volume of snake imports (**a**) and exports (**b**) from 1975 to 2022, measured in Whole Organism Equivalents (WOEs) and categorized by source (wild-sourced vs. captive-bred). Inset (g) highlights the export trends from 1995 to 2022. Total volume (WOEs) of wild-sourced snakes imported from (**c**) and exported to (**d**) major CITES parties, color-coded by taxonomic family. Trade in live individuals (number of individuals) imported from (**e**) and exported to (**f**) major CITES parties, categorized by taxonomic family. Inset (h) provides a magnified view of smaller export markets for live snakes. DE: Germany; ES: Spain; FR: France; GB: United Kingdom; GH: Ghana; HK: Hong Kong, China; ID: Indonesia; IT: Italy; JP: Japan; KH: Cambodia; KR: Republic of Korea; LA: Laos; MO: Macao, China MX: Mexico; MY: Malaysia; NL: Netherlands; SG: Singapore; TG: Togo; TW: Taiwan, China; US: United States of America; VN: Vietnam.

Wild-sourced individuals dominated China’s snake trade between 1975 and 2023, with significant trade peaks recorded around 2000 and in the mid-2010s (Figure 2a,b). Since 2010, the proportion of captive-bred individuals in both imports and exports has risen year on year; notably, the volume of captive-bred imports surpassed that of wild-sourced imports for the first time in 2014 (Figure 2a). Source composition varied significantly across families: in import records, snakes of the families Elapidae (89.5%) and Colubridae (78.5%) were primarily wild-sourced, whereas snakes of the family Viperidae were predominantly captive-bred (98.4%). In export records with reported source categories, the vast majority of colubrid (99.5%) and elapid (99.7%) snakes were reported as being wild-sourced (Figure S1).

Regarding conservation status, wild-sourced exports were highly taxonomically concentrated. Species assessed as “Least Concern” (LC) on the IUCN Red List accounted for more than 420,000 WOEs. The common cobra (*Naja naja*) was the most frequently exported wild-sourced species, comprising 67.7% of all identified wild-sourced exports, followed by *M. reticulatus*. Vulnerable (VU) species were also present in wild-sourced export records, including the beauty ratsnake (*Elaphe taeniura*), the Chinese cobra (*Naja atra*), and the king cobra (*Ophiophagus hannah*) (Table 3). Furthermore, a significant volume of wild-sourced *N. naja* was recorded as exported from China in the CITES database, despite IUCN distribution data indicating that this species has no natural distribution within China’s borders.

Geographical analysis identified Hong Kong (Special Administrative Region, SAR) as the primary trading partner for wild-sourced snakes, whereas Vietnam, Indonesia, and Malaysia served as the core supplier countries (Figure 2c,d). The live snake trade exhibited distinct regionalization, with 98.9% of live imports originating from Southeast Asian nations, among which Vietnam provided the largest supply (Figure 2e). Market demand for snake families varied significantly by destination: live exports to Hong Kong (65.6%), Macau SAR (61.1%), and Italy (100%) were dominated by colubrid snakes; exports to Taiwan (85.6%) and Japan (96.7%) primarily involved elapid snakes, whereas snakes of the family Boidae accounted for 95.0% of the live exports recorded for the United States market (Figure 2f and Figure S2).

### 3.3. Venomous Snake Trade

**Figure 3:**
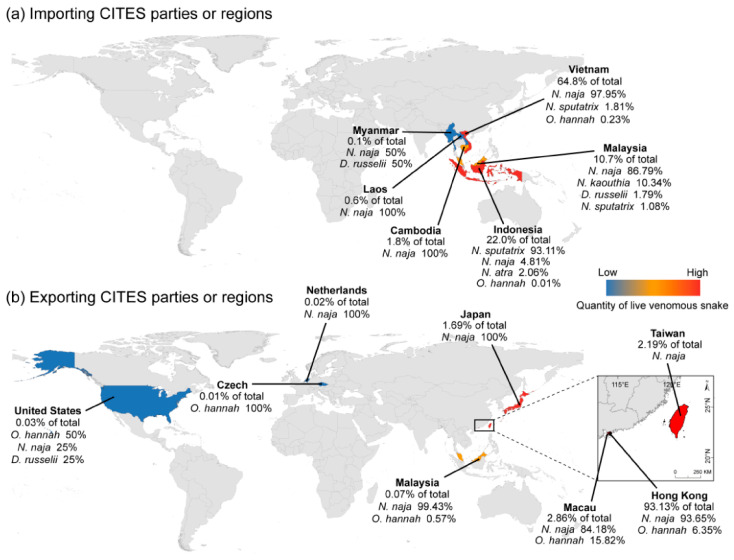
Global distribution of CITES parties involved in the trade of live venomous snakes in China.

The live venomous snake trade occupies a significant position within China’s snake trade network, characterized by highly differentiated geographic patterns for imports and exports. Data indicate that live venomous snakes accounted for 20.2% (330,684 WOEs) of China’s total live snake imports and 34.6% (270,423 WOEs) of the country’s exports. Imports exhibited high geographic concentration, primarily originating from Vietnam (64.8%), Indonesia (22.0%), and Malaysia (10.7%), with key species including *N. naja*, the Indonesian cobra (*Naja sputatrix*), and *O. hannah* (Figure 3a). In contrast, although the export network for live venomous snakes was broader, destinations were highly skewed toward specific regions: 93.1% of live venomous snakes were exported to Hong Kong, followed by Macau (2.8%) and Taiwan (2.2%), China. At the species level, *N. naja* accounted for 93.6% of venomous snake exports, with 93.2% of this volume flowing to Hong Kong. *Ophiophagus hannah* was the second-largest exported venomous species, with 92.5% of its export volume similarly destined for Hong Kong (Figure 3b).

### 3.4. The Role Transformation of China’s Snake Trade

**Figure 4:**
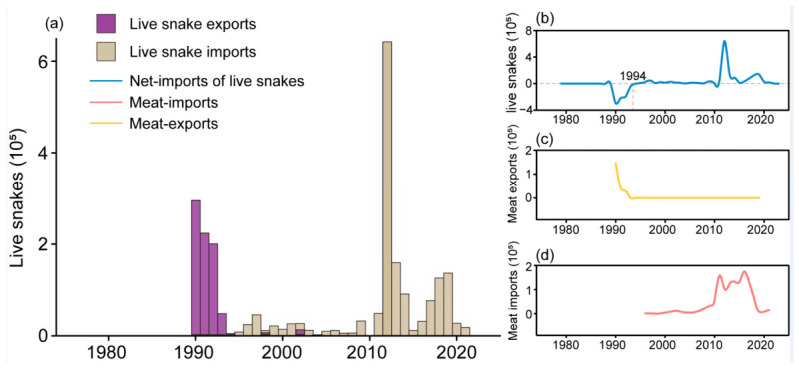
Live snake and snake meat trade in China since 1975, showing the annual number of live individuals exported (purple) and imported (beige) (**a**), annual net imports of live snakes (**b**), annual exports of snake meat in kg (**c**), and annual imports of snake meat in kg (**d**).

Over the past three decades, China’s role in the global snake trade underwent a fundamental transformation from a primary exporter to a primary importer. In the early 1990s, China was a major global supplier of live snakes, reaching a peak annual export volume of 290,000 individuals. However, since 1993, live snake exports have plummeted and have subsequently remained at relatively low levels (Figure 4). Simultaneously, live snake imports began to rise with fluctuations in the mid-1990s, reaching a historical high in 2012 and maintaining a volume of over 100,000 individuals annually thereafter. The snake meat trade exhibited a similar trend: exports were concentrated in the early 1990s, while trade flows in the 21st century transitioned to an import-dominated model, with total import volumes far exceeding total exports (Figure 4).

## 4. Discussion

### 4.1. China’s Snake Trade and Farming Models Driven by Culinary-Medicinal Traditions

Our study reveals that China’s snake trade patterns diverge significantly from global mainstream models in terms of species preference. Unlike the international high-end leather market’s reliance on large pythonid snakes such as *M. reticulatus* [18], the Chinese market favors medium-sized colubrid snakes such as *P. mucosa* and the king ratsnake *Elaphe carinata*. This distinctive pattern is deeply rooted in China’s profound culture of “medicine and food homology,” particularly the massive demand for snake meat (e.g., snake soup) in South China [5]. Following the Reform and Opening-up policy, coupled with the advancement of logistics, snake consumption has expanded from a local tradition to a nationwide phenomenon, with an estimated annual trade volume of 7 to 9 million kilograms [21]. This internal driver, centered on culinary and medicinal use, transformed China from a primary resource supplier in the 1990s into a massive consumption hub that sustains the global snake trade in the 21st century (Figure 4).

The growing demand for meat directly catalyzed the boom in China’s snake farming industry. Snake ranching and breeding, especially in China and Vietnam, have evolved into a substantial industry [15]. Research indicates that closed-cycle captive breeding for snakes like pythons is biologically and economically feasible [15,22], providing a precedent for farming colubrid snakes to meet domestic meat demand. Theoretically, efficient captive breeding could alleviate harvest pressure on wild populations. However, systematic assessments of the conservation efficacy of wildlife farming suggest that the reality is far more complex than the theory, with challenges such as a lack of reliable assessment frameworks and robust evidence; outcomes are highly dependent on specific species, geographic contexts, and market dynamics [23]. Our preliminary investigations observed that the industry currently exhibits a “small-scale, fragmented, and poorly regulated” pattern. Practitioners often rely on experience-based, extensive management, leading to prominent environmental and biosecurity risks. A core challenge is that the stock (parental individuals or eggs) for many so-called “captive-bred” individuals may still originate from the wild, making farms susceptible to becoming legal veneers for “laundering” illegal wild resources. For instance, in the python trade, studies have discovered that up to 90% of individuals labeled as “legally farmed” for export were actually wild-caught [16], a practice that exacerbates the depletion of wild populations [24].

Crucially, this study highlights a key contradiction in China’s snake trade: while the contribution of captive-bred sources in both imports and exports has significantly increased since 2010 (Figure 2a,b), wild-sourced trade has persisted throughout history, resulting in a dual-supply model where “captive” and “wild” sources coexist (Figure S1). This divergence is particularly stark across different snake families: while pythonid snakes have largely transitioned to captive breeding, a large number of colubrid and elapid snakes continue to rely on wild harvest. This clearly indicates two parallel supply chains within China’s snake trade: one primarily based on captive breeding for the domestic market, and another persistently reliant on wild capture for specific processing, re-export, or domestic consumption needs. This market segmentation, where farmed products cannot fully substitute for wild ones, may stem from a strong consumer preference for “wild-caught” products—a demand-side driver considered key to the persistence of wild harvesting [25,26].

The “wildlife ban” implemented following the COVID-19 pandemic has had a disruptive impact on the meat-oriented snake farming industry [27]. Due to the differential management of terrestrial versus aquatic wildlife and the complexities in defining the boundary between “medicinal” and “culinary” use, the previously legal edible-snake farming industry was suddenly pushed into a “grey area.” Although this stringent regulation significantly reduced legal trade volumes in the short term (Figure 4), it also resulted in immense economic losses for the practitioners running large-scale farms. A deeper concern is that, without scientific guidance for industrial transition, aggressive administrative bans may drive the massive demand for snake consumption into more concealed underground markets [28], thereby increasing regulatory difficulty and triggering new public health risks [23].

Spanning nearly fifty years of evolution, China’s snake trade has progressed through three stages: traditional small-scale utilization, meat-driven industrial farming following the Reform and Opening-up, and strict policy regulation following COVID-19. This trajectory reflects a profound tug-of-war between traditional cultural demands and modern public health management. The fluctuations in the trade of key species like *P. mucosa* are not only a reflection of market economic laws but also a microcosm of China’s search for a scientific balance between ecological conservation, cultural heritage, and livelihood security.

### 4.2. Distinctive Geographical Patterns and Potential Risks of Live Venomous Snake Trade

Our study confirms China’s pivotal position as a hub in the global live venomous snake trade network. Driven by the traditional belief that “greater toxicity confers higher tonic efficacy”, venomous snakes are widely used in China for high-end culinary and medicinal purposes [21]. On one hand, massive market demand attracts substantial volumes of *N. naja* and *N. sputatrix* from Southeast Asia (Vietnam, Indonesia, and Malaysia). On the other hand, as a key exporter, China exhibits a striking degree of geographic concentration: over 93% of live venomous snakes—primarily meat-oriented species such as *N. kaouthia*, *N. atra*, and *O. hannah*—flow into Hong Kong (Figure 3). This finding validates Hong Kong’s specialized role as a long-standing, stable transit hub and terminal market for China’s snake trade [1,5].

This highly concentrated trade model entails severe cascading risks. First, the trade chain’s heavy reliance on wild capture exposes personnel across the entire spectrum—from hunters to consumers—to fatal snakebite risks [18]. Second, high-density captive environments and the mixing of species create ideal conditions for interspecies pathogen transmission and the introduction of invasive alien parasites [10,29]—risks for which the COVID-19 pandemic has served as a critical wake-up call [30]. Exotic parasites can be introduced into new ecosystems via the snake trade, potentially dealing devastating blows to native species [10,31]. Furthermore, trade-induced ecological perturbations can indirectly trigger public health crises; for instance, the invasion of exotic pythons has led to a sharp decline in local mammals, forcing pathogen hosts to shift toward humans and thereby increasing zoonotic risks [32].

The risk inherent in wildlife trade is essentially a “collective action problem”: traders reap the economic benefits, while the resulting public health risks are externalized to society as a whole [33]. Compared to “one-size-fits-all” trade bans, extending established food safety management systems to the wildlife trade appears more feasible [34]. For example, the Hazard Analysis Critical Control Point (HACCP) system, widely used in the livestock industry, could be implemented. By scientifically identifying “critical control points” across the entire chain—from capture and transport to consumption—and establishing traceable risk monitoring and prevention mechanisms, it is possible to systematically mitigate the ecological and health risks associated with the trade system [34].

### 4.3. Limitations of CITES Trade Data and Methodological Considerations

The CITES Trade Database serves as the authoritative foundation for understanding global snake supply–demand dynamics, monitoring the scale of species utilization, and assessing regulatory compliance [35]. However, its interpretation requires caution, as it only captures legal annual reports and reflects dynamics within the regulated scope. Of the more than 3700 snake species globally, fewer than 200 are currently listed in the CITES Appendices, meaning that the vast majority of trade remains outside the purview of international regulatory oversight [12,17]. This limited coverage poses a significant challenge to accurately assessing the global sustainability of snake resources [4,36].

The most conspicuous finding in this study is the substantial volume of *N. naja* recorded as wild-sourced exports from China, despite the species having no natural distribution within Chinese borders [37]. This anomaly strongly suggests widespread misreporting; these individuals are highly likely to be the native *N. atra*, a species with a deep-rooted taxonomic history with *N. naja* [17]. Such data inconsistencies are not isolated incidents; they represent a core challenge in global trade regulation. The root cause lies in the resource and capacity constraints faced by signatory parties regarding precise species identification, coding protocols, and reporting compliance [20,38,39], which leaves a significant portion of trade records in a state of ambiguity [3].

The substantial gap between importer- and exporter-reported data is another systemic feature of CITES records [38]. Since parties are not mandated to report import data for CITES Appendix II species—which comprise the bulk of the snake trade—import records systematically underestimate actual trade volumes. Conversely, while export data are more comprehensive, they may overestimate trade volumes, as they often reflect “permitted quantities” rather than “actual trade volumes” [20]. Furthermore, the current database lacks unique batch IDs that span both borders and years, making it technically almost impossible to track re-export pathways or avoid double-counting [18].

It must be emphasized that officially recorded seizure data, due to poor reporting compliance, represent merely a highly conservative “tip of the iceberg” with respect to illegal trade [40]. Facing multifaceted challenges—including identification biases, reporting discrepancies, and ambiguities in units and terms—this study adopted a prudent data comparison strategy. We aggregated only comparable import and export data and implemented targeted classification corrections for anomalous species information. This methodological rigor aims to minimize interpretive bias, ensuring that the trade trends and patterns identified remain scientifically robust despite the inherent limitations of the data.

## 5. Conclusions

By systematically analyzing CITES trade data from 1975 to 2023, this study provides a comprehensive portrait of China’s temporal dynamics and its conservation implications within the global snake trade network. Our findings confirm that China, together with the United States and Japan, ranks among the top three legal wildlife trading nations globally [3]. China plays a multifaceted role: it serves as both a global processing and export center for snake skin products and a critical hub for the live snake trade, particularly for venomous species. This pattern, driven by a robust domestic culinary and medicinal market, has exhibited remarkable stability over the past half-century. Such sustained and concentrated trade pressure has become a core driver of species depletion [2], suggesting that habitat protection alone is no longer sufficient; instead, there is an urgent need for more stringent trade regulation and scientifically rigorous sustainability assessment frameworks.

Future research and conservation actions should focus on three critical areas. First, there is an urgent need to utilize molecular tools (e.g., genetic sourcing and barcoding) to clarify the true species identity of traded individuals, particularly to reveal the taxonomic reality and potential ecological risks behind records labeled as “*Naja naja*”. Second, field population monitoring should be strengthened for high-volume traded species such as *P. mucosa*, *E. carinata*, and *N. atra* to objectively evaluate the sustainability of captive breeding and its cascading effects on ecosystems [26]. Finally, the highly stable “Mainland China–Hong Kong” venomous snake trade chain must be scrutinized to identify end uses and re-export networks, providing scientific early warnings about associated public health and biosecurity risks.

The conservation efficacy of snake farming represents a double-edged sword: while it supports rural livelihoods, it rarely generates positive incentives for habitat conservation if entirely decoupled from wild populations and may even cause negative impacts through market competition [22]. Therefore, future governance should adopt an integrated strategy that explicitly couples controlled captive breeding with sustainable wild population management and habitat conservation goals. This requires the establishment of mandatory provenance audits and traceability systems, the promotion of standardized farming certifications, and the implementation of humane slaughter standards [22]. Such measures will help strike a more resilient balance between ecological conservation, cultural heritage, and livelihood security.

## Figures and Tables

**Table 1:** The quantity of snake products (exported/imported) classified by CITES trade term and snake family. All units are in WOEs.

Term	Family								Total
	Acrochordidae	Boidae	Colubridae	Elapidae	Hydrophiidae	Pythonidae	Viperidae	Xenopeltidae	
Bodies	0/0	12/0	391/7161	36/0	0/0	36/4	0/1	0/0	475/7166
Live	0/0	4603/2312	506,347/1,276,830	270,403/329,954	0/0	1147/23,502	20/730	2/0	782,522/1,633,328
Skeletons	0/0	0/0	0/0	0/0	0/0	2/2	0/0	0/0	2/2
Skins	418/8853	47/550	5,341,919/1,085,557	802,953/182,539	708/0	244,281/1,168,822	9/1593	0/1500	6,390,335/2,449,414
Skulls	0/0	0/0	0/0	0/0	0/0	0/0	0/0	0/0	0
Tails	0/0	0/0	0/0	0/0	0/0	12/0	0/0	0/0	12
Trophies	0/0	0/0	0/0	0/0	0/0	0/0	0/0	0/0	0
Specimens	0/0	2/0	661/90,208	71/1	0/0	23/10,823	0/387,163	0/0	757/488,195
Egg	0/0	0/0	0/0	1/0	0/0	0/18,000	0/0	0/0	1/18,000
Bladders	0/0	1/0	0/0	3/0	0/0	2/124	0/0	0/0	6/124
Total	418/8853	4665/2862	5,849,318/2,459,756	1,073,467/512,494	708/0	245,503/1,221,277	29/389,487	2/1500	7,174,110/4,596,229

**Table 2:** Top-traded species recorded in the CITES Trade Database per snake family (max. 10 species per family).

Family	Species	WOEs		Total
		Export (%)	Import (%)	
Acrochordidae	*Acrochordus javanicus*	418 (0.01%)	8853 (0.19%)	9271
Boidae	*Corallus* spp.	4556 (0.06%)	0 (0%)	4556
	*Boa constrictor*	6 (<0.01%)	1757 (0.04%)	1763
	*Eunectes notaeus*	0 (0%)	427 (0.01%)	427
	*Boa* spp.	60 (<0.01%)	123 (<0.01%)	183
	*Epicrates cenchria*	4 (<0.01%)	131 (<0.01%)	135
	*Lichanura trivirgata*	0 (0%)	101 (<0.01%)	101
	*Eunectes murinus*	10 (<0.01%)	56 (<0.01%)	66
	*Corallus hortulana*	0 (0%)	51 (<0.01%)	51
	*Chilabothrus angulifer*	25 (<0.01%)	1 (0%)	26
Colubridae	*Ptyas mucosa*	4,078,042 (56.84%)	1,927,958 (41.95%)	6,006,000
	*Elaphe carinata*	968,843 (13.50%)	229,000 (4.98%)	1,197,843
	*Coelognathus radiatus*	742,913 (10.36%)	193,515 (4.21%)	936,428
	*Homalopsis buccata*	40,696 (0.57%)	47,900 (1.04%)	88,596
	*Cerberus rynchops*	899 (0.01%)	33,127 (0.72%)	34,026
	*Ptyas korros*	35 (<0.01%)	23,000 (0.5%)	23,035
	*Elaphe taeniura*	17,777 (0.25%)	0 (0%)	17,777
	*Fowlea piscator*	11 (<0.01%)	5255 (0.11%)	5266
	*Ptyas dhumnades*	95 (<0.01%)	0 (0%)	95
Elapidae	*Naja naja*	1,033,904 (14.41%)	372,508 (8.11%)	1,406,412
	*Naja sputatrix*	7 (<0.01%)	133,853 (2.91%)	133,860
	*Ophiophagus hannah*	37,523 (0.52%)	980 (0.02%)	38,503
	*Naja kaouthia*	3 (<0.01%)	3653 (0.08%)	3656
	*Naja atra*	2022 (0.03%)	1500 (0.03%)	3522
	*Hydrophis curtus*	708 (0.01%)	0 (0%)	708
	*Micrurus diastema*	4 (<0.01%)	0 (0%)	4
	*Naja* spp.	4 (<0.01%)	0 (0%)	4
Homalopsidae	*Myrrophis chinensis*	4 (0%)	0 (0%)	4
Pythonidae	*Malayopython reticulatus*	187,439 (2.61%)	750,379 (16.33%)	937,818
	*Python bivittatus*	42,747 (0.6%)	414,255 (9.01%)	457,002
	*Python brongersmai*	4516 (0.06%)	18,532 (0.4%)	23,048
	*Python curtus*	6763 (0.09%)	11,913 (0.26%)	18,676
	*Python regius*	46 (<0.01%)	12,315 (0.27%)	12,361
	*Python sebae*	0 (0%)	6194 (0.13%)	6194
	*Python molurus*	1616 (0.02%)	4516 (0.1%)	6132
	*Python breitensteini*	1501 (0.02%)	2812 (0.06%)	4313
	*Simalia amethistina*	800 (0.01%)	0 (0%)	800
	*Morelia spilota*	0 (0%)	128 (<0.01%)	128
Viperidae	*Daboia russelii*	29 (<0.01%)	389,486 (8.47%)	389,515
	*Crotalus durissus*	0 (0%)	1 (<0.01%)	1
Xenopeltidae	*Xenopeltis unicolor*	2 (<0.01%)	1500 (0.03%)	1502

**Table 3:** Export quantities of wild-sourced snake species recorded in the CITES Trade Database, categorized by IUCN Red List status.

IUCN Status	Species	Quantity of Shipped WOE
Least Concern	*Naja naja*	298,938 (67.67%)
	*Malayopython reticulatus*	98,504 (22.30%)
	*Coelognathus radiatus*	14,573 (3.30%)
	*Python curtus*	6763 (1.53%)
	*Python brongersmai*	4516 (1.02%)
	*Python bivittatus*	3255 (0.74%)
	*Elaphe carinata*	2835 (0.64%)
	*Python breitensteini*	1500 (0.34%)
	*Acrochordus javanicus*	258 (0.06%)
	*Chilabothrus angulifer*	25 (<0.01%)
	*Daboia russelii*	20 (<0.01%)
	*Morelia viridis*	3 (<0.01%)
	*Boa constrictor*	2 (<0.01%)
	*Fowlea piscator*	1 (<0.01%)
	*Naja kaouthia*	1 (<0.01%)
	*Naja sputatrix*	1 (<0.01%)
Near Threatened	*Python molurus*	51 (~0.01%)
	*Ptyas korros*	35 (~0.01%)
	*Python regius*	6 (<0.01%)
Vulnerable	*Elaphe taeniura*	6983 (1.58%)
	*Naja atra*	2000 (0.45%)
	*Ophiophagus hannah*	1510 (0.34%)

## Data Availability

Data are contained within the article.

## Supplementary Materials

The following supporting information can be downloaded at: https://www.mdpi.com/article/10.3390/ani16111624/s1.

## Abbreviations

The following abbreviations are used in this manuscript:

CITES	The Convention on International Trade in Endangered Species of Wild Fauna and Flora
HACCP	The Hazard Analysis Critical Control Point
IUCN	International Union for Conservation of Nature
LC	Least Concern
SAR	Special Administrative Region
UNEP-WCMC	UN Environment Programme World Conservation Monitoring Centre
VU	Vulnerable
WOEs	Whole Organism Equivalents
