# Supplementary material for: Patterns and Risks of China’s Snake Trade Driven by Medicinal and Culinary Traditions
Source: Animals (Basel). 2026 May 27;16(11):1624. doi: 10.3390/ani16111624 (PMC13255723; doi:10.3390/ani16111624)
Supplement: Supplementary file 1 [file animals-16-01624-s001.zip › Tables_S/TableS1.pdf]

Table S1. Summary of the species with updated Latin names.

Previous species name	Updated species name
Elaphe radiata	*Coelognathus radiatus*
Xenochrophis piscator	*Fowlea piscator*
Corallus hortulanus	*Corallus hortulana*
Ptyas mucosus	*Ptyas mucosa*
Zaocys dhua	*Ptyas dhumnades*
Enhydris chinensis	*Myrrophis chinensis*
Lapemis curtus	*Hydrophis curtus*
Morelia amethistina	*Simalia amethistina*
