# Supplementary material for: Patterns and Risks of China’s Snake Trade Driven by Medicinal and Culinary Traditions
Source: Animals (Basel). 2026 May 27;16(11):1624. doi: 10.3390/ani16111624 (PMC13255723; doi:10.3390/ani16111624)
Supplement: Supplementary file 1 [file animals-16-01624-s001.zip › Tables_S/TableS2.pdf]

Table S2. Categorization of raw CITES source codes into wild- and captive-sourced classes and excluded source codes.

**Wild**	W	Specimens taken from the wild
	U	Source unknown
	X	Specimens taken in "the marine environment not under the jurisdiction of any state"
	R	'Ranched' specimens are those taken as eggs or juveniles from the wild, where they Would otherwise have had a very low survival probability, and reared in a controlled environment, often with the release of some of the offspring back into the wild
**Captive**	C	An animal is 'captive-bred' when it is produced in a controlled environment under certain conditions, including that a) reproduction took place in that environment, b) the breeding stock was established in line with the provisions of CITES and national laws without detriment to the survival of the species in the wild, c) that the breeding stock is maintained without the introduction of specimens from the wild, except for The occasional additional of animals to prevent/alleviate deleterious inbreeding or exceptionally for use as breeding stock, (where the CITES Scientific Authority advises this is non-detrimental) and d) the breeding stock has either produced offspring of second-generation (F2) or subsequent generations in a controlled environment, or be managed in a manner that has been demonstrated to be capable of reliably producing F2 offspring. Additional details in CITES Resolutions Conf. 10.16 (Rev.)
	D	Appendix-I animals bred in captivity and Appendix-I plants artificially propagated for Commercial purposes. Additional details in CITES Resolutions Conf. 12.10 (Rev. CoP15).
	A	Plants that are artificially propagated, when they are grown under controlled conditions and have been derived from cultivated parental stock
	F	Animals born in captivity (F1 or subsequent generations) that do not fulfil the definition of 'bred in captivity' (Source code 'C').
**Excluded**	I	Confiscated or seized specimens
	O	Pre-convention specimens
	Blank	Source unreported
