# Supplementary material for: Patterns and Risks of China’s Snake Trade Driven by Medicinal and Culinary Traditions
Source: Animals (Basel). 2026 May 27;16(11):1624. doi: 10.3390/ani16111624 (PMC13255723; doi:10.3390/ani16111624)
Supplement: Supplementary file 1 [file animals-16-01624-s001.zip › Tables_S/TableS3.pdf]

Table S3. Quantity of snakes (exported/imported) by purpose of trade and family. All units are in WOEs

Purpose	Acrochordidae	Boidae	Colubridae	Elapidae	Hydrophiidae	Pythonidae	Viperidae	Xenopeltidae	Total
Breeding in captivity or artificial propagation	0/0	0/70	0/73000	0/11100	0/0	40/291	0/100	0/0	40/84561
Educational	0/0	3/3	9/10	3/1	0/0	128/135	0/1	0/0	143/150
Hunting trophy	0/0	0/0	206/0	0/0	0/0	0/0	0/0	0/0	206/0
Law enforcement / judicial / forensic	0/0	0/123	0/228	0/290	0/0	0/0	0/0	0/0	0/641
Medical (including biomedical research)	0/0	1/0	0/0	0/0	0/0	0/0	0/18200	0/0	1/18200
Reintroduction or introduction into the wild	0/0	0/0	0/1	0/0	0/0	0/242	0/0	0/0	0/243
Personal	0/0	35/2	1/0	10/0	0/0	457/272	0/0	0/0	503/274
Circus or travelling exhibition	0/0	2/4	0/0	6/6	0/0	66/114	0/0	0/0	74/124
Scientific	0/0	3/3	1/0	0/0	0/0	6/26	0/0	0/0	10/29
Commercial	258/8853	4596/2551	3593557/2211182	989241/472013	0/0	227438/1214393	20/371186	0/1500	4815110/4281678
Zoo	0/0	1/94	0/4	0/1000	0/0	1165/106	0/0	0/0	1166/1204
Missing	160/0	24/12	2255544/175331	84207/28084	708/0	16203/5698	9/0	2/0	2356857/209125
Total	418/8853	4665/2862	5849318/2459756	1073467/512494	708/0	245503/1221277	29/389487	2/1500	7174110/4596229
