# Supplementary figures and images for: Patterns and Risks of China’s Snake Trade Driven by Medicinal and Culinary Traditions
Source: Animals (Basel). 2026 May 27;16(11):1624. doi: 10.3390/ani16111624 (PMC13255723; doi:10.3390/ani16111624)

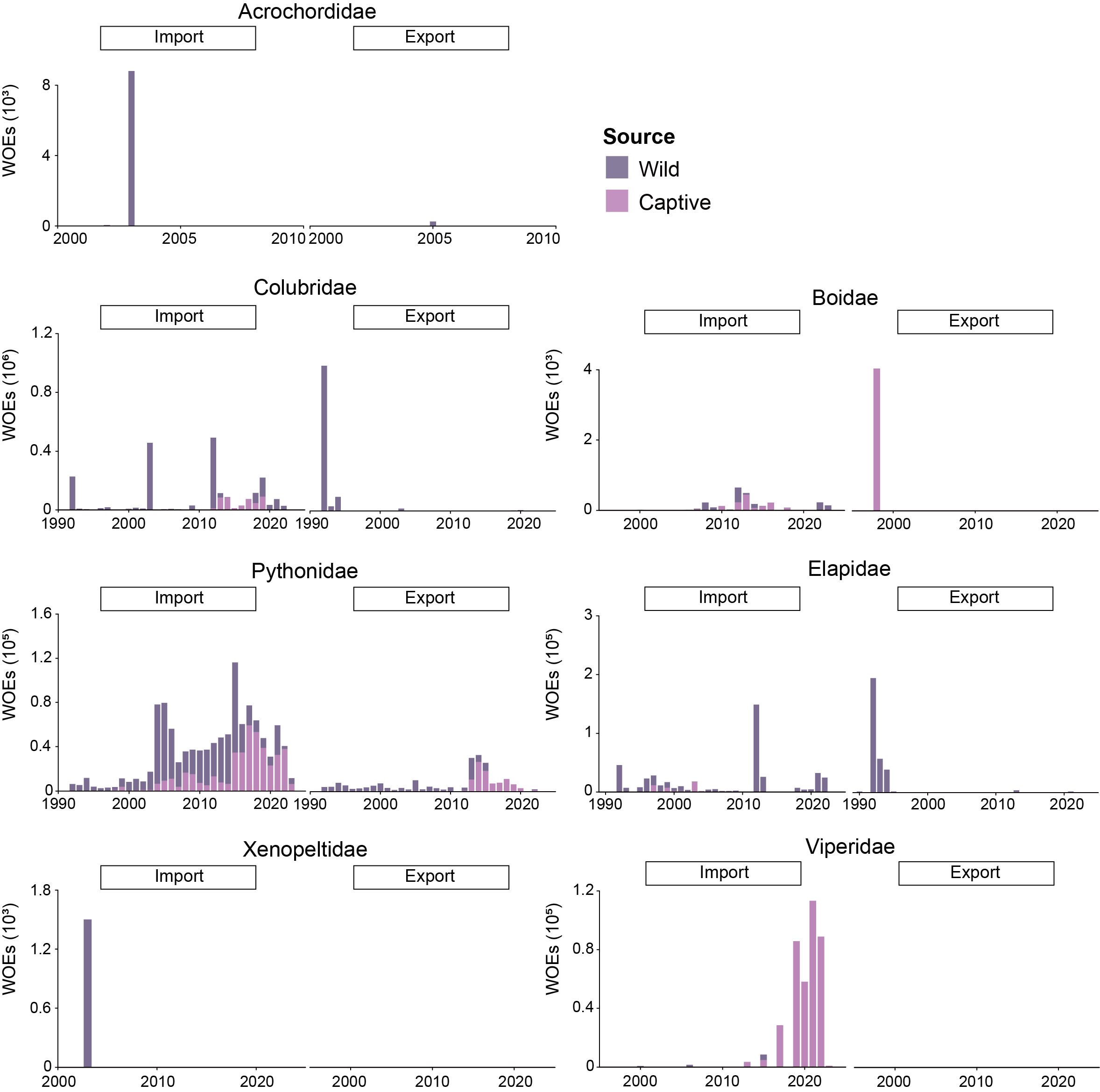

Supplement: Supplementary file 1 [file animals-16-01624-s001.zip › Figures_S/FigureS1.tif]

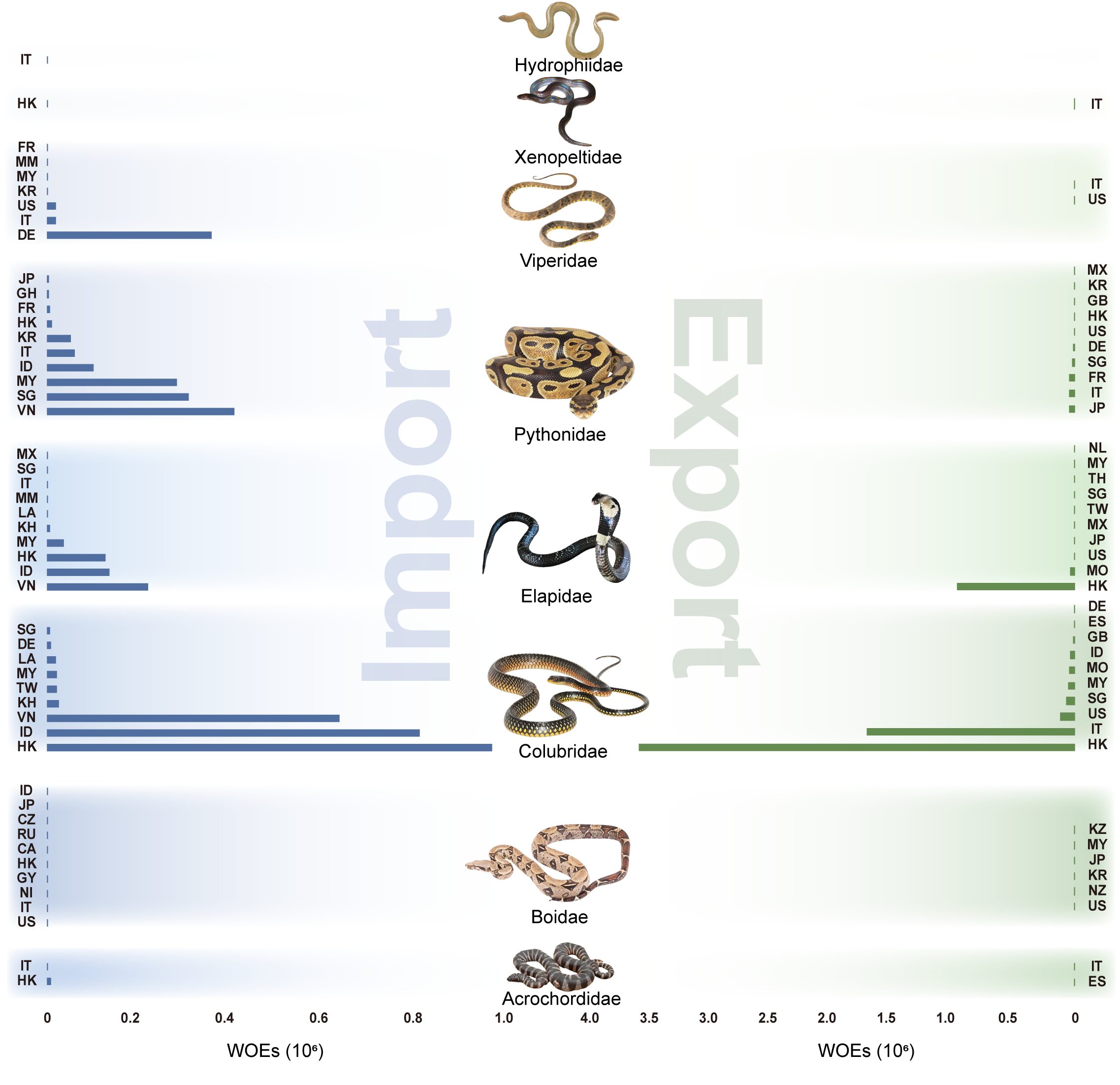

Supplement: Supplementary file 1 [file animals-16-01624-s001.zip › Figures_S/FigureS2.tif]
